# Virtual Reality as RESPITE: Relief Exploration for Sickle Pain Through Interventions Using Technology Engagement: A Hospital-Based Observational Study

**DOI:** 10.1007/s11606-025-09812-z

**Published:** 2025-08-15

**Authors:** Nitin Vidyasagar, May Nguyen, Andrea Bundy, Nabil Abou Baker, Dima Kenj Halabi, Vineet M. Arora, David O. Meltzer, Valerie G. Press

**Affiliations:** 1 Pritzker School of Medicine, The University of Chicago, Chicago, USA; 2 Department of Medicine, University of Chicago, Chicago, USA; 3 Center for Transformative Care, University of Chicago Medicine, Chicago, USA; 4 Department of Medicine and Pediatrics, University of Chicago, Chicago, USA

**Keywords:** virtual reality, sickle cell, pain, health literacy, electronic health literacy

## Abstract

**Background:**

The opioid epidemic, side effects from acute and chronic opioid use, and low utilization of preventive medications for sickle cell disease (SCD) necessitate the development of novel strategies to manage painful vaso-occlusive episodes. Virtual reality (VR) has shown promise for pediatric patients with SCD; however, interest in VR among adults with SCD is under examined. Ensuring equitable access to VR-based interventions requires understanding of factors that determine patients’ interest in VR.

**Objective:**

To assess interest, preferences, and predictors regarding interest in VR-based interventions to manage SCD-related pain.

**Design:**

Cross-sectional, observational, single-site study conducted between 2023 and 2024.

**Participants:**

Hospitalized, English-speaking, had a SCD diagnosis, and aged ≥ 18 years.

**Main Measures:**

The primary outcome was participants’ interest in using VR. General health literacy (HL) and electronic health literacy (eHL) were assessed using Brief Health Literacy Screen and eHealth Literacy Scale, respectively, to compare differences between participants interested versus uninterested in using VR to manage pain. Participants also indicated preferred gaming genres and barriers to VR use.

**Key Results:**

Among 40 participants (80% response rate), most self-identified as Black (95%) and male (58%); 43% had low HL; and 13% had low eHL. Most (85%) participants indicated interest in using VR for pain management (*n* = 34/40). Participants uninterested in using VR were more likely to have low HL (100% vs. 33%; *p* = 0.002) and low eHL (67% vs. 3%; *p* < 0.001) than participants interested in VR. Skill development (82%; e.g., learning how to rock-climb) and adventure (82%) genres were most preferred among interested participants. Cost (65%) and access to VR devices (35%) were cited as notable barriers.

**Conclusions:**

The present study identifies that people with SCD regard VR as a promising intervention for pain management. Future studies include designing equitably accessible VR interventions and assessing efficacy, paying attention to factors like electronic and general health literacy.

**Supplementary Information:**

The online version contains supplementary material available at 10.1007/s11606-025-09812-z.

## INTRODUCTION

Among the 90,000 Americans living with sickle cell disease (SCD), many experience acute episodes of pain due to vaso-occlusive episodes (VOE) who may then subsequently develop chronic pain.^1^ Pain from VOE leads to hospitalizations and emergency department visits and represents the majority of SCD-related care costs for patients.^2^

Acute VOE pain is managed through treatments such as opioids. Preventative and curative pain therapies, including hydroxyurea, stem cell transplants, and gene therapy, are often underutilized due to cost, care-transition difficulties, and lack of clinician knowledge.^3,4^ Optimizing innovative pain regimens is critical for many reasons, including hyperalgesia and opioid addiction. Recent qualitative studies identified that patients with SCD face barriers to obtaining opioid prescriptions, including increased restrictive prescribing habits, increased monitoring, difficulty in filling prescriptions in pharmacies, and a perception that physicians’ exclusive focus on reducing pain medication use affected medical care.^5,6^

Non-pharmacological forms of pain management, including technology and art, may be used by adults with SCD for pain management either primarily or as a supplement to pharmacologic therapies.^7^ Virtual reality (VR) is an innovative approach that may reduce pain through distraction and immersion. Studies of use for chronic pain demonstrate VR’s effectiveness, including for decreased pain among adults who survived breast cancer (decreases in pre/post mean Defense and Veterans Pain Rating Scale scores of 7.32 vs. 0.33; *p* < 0.001)^8^ and adults with chronic back pain (VR versus sham VR reduction of pain intensity via Visual Analog Scale; *p* = 0.001.^9^ Promisingly, VR has been demonstrated to reduce VOE pain in pediatric patients with SCD.^10^ Critically, studies of VR use among *adult* patients with SCD are lacking, particularly in the hospital setting, which may ameliorate pain in adults with SCD hospitalized for VOEs. Furthermore, the efficacy of VR-based management of both acute and chronic pain from VOE in adults remains unstudied. Therefore, the first step in devising these interventions is to identify the perspectives on barriers and facilitators to VR use among adults living with SCD. Without these perspectives, patients may not engage with, or have equitable access to, these technology-based interventions, due in part to the digital divide.^11–13^ Barriers such as cost, access to devices, and digital health literacy may be barriers that impact outpatient and home use of interventions.

While technology-based solutions such as VR can expand and enhance care for patients, there are well-documented concerns about widening health disparities when implementing technology-based interventions.^14^ Therefore, bridging this divide necessitates understanding factors that impact interest in access to using technology-based solutions such as VR. Critical to this understanding are two important factors to individuals’ uptake and use of interventions, namely health literacy (HL) for overall engagement and electronic health literacy (eHL), related to technology engagement. Both HL and eHL have shown associations regarding lower engagement in areas such as patient portals, telehealth, and fitness apps.^12,13,15^

Our study, Insights into Relief Exploration for Sickle Pain through Interventions using Technology Engagement (RESPITE), assessed the interest, preferences, and barriers to using VR among adult participants with SCD, including associations with HL and eHL.

## METHODS

### Study Design

This cross-sectional, observational study was conducted among adult inpatients at the University of Chicago Medicine. Eligibility included hospitalized on general medicine services; diagnosis of SCD; age 18 years or older; and English speaking. In preparation for developing an inpatient VR intervention for adults with active VOE, we aimed to identify baseline needs and preferences among adults with VOE who were hospitalized. Our Institutional Review Board approved this protocol (IRB 16–0763-AM048); this study follows the Strengthening the Reporting of Observational Studies in Epidemiology (STROBE) reporting guideline.^16^

### Measures

The primary outcome variable was self-reported interest in using VR for SCD pain relief, captured through the following responses: yes, no, don’t know, and refused. Interest was recoded as a binary variable to capture interest (yes) and disinterest (no, don’t know and refused). Interested participants were asked to select all that apply for barriers to using VR and preferences for VR genres (adventure, puzzle-solving games, meditation, skill-development). Our survey was administered by an interviewer either in person or by phone, reducing potential biases encountered by internet surveys, which might be more accessible to people with greater technology access and capabilities.

For associations of primary outcomes with HL and eHL, we used the validated Brief Health Literacy Screen (BHLS) and 8-item eHealth literacy scale (eHEALS) with both a binary and continuous evaluation of the variables (binary: low HL: ≤ 2 on any of three items; low eHL: < 26 [range: 8–40]) ^17,18^ Descriptive statistics, Fisher’s exact or Pearson’s chi-squared and Student’s two-tailed *t*-tests, were performed to analyze differences between participants interested versus uninterested in using VR. Differences in composite eHEALS and BHLS scores were analyzed using Mann–Whitney *U* test and Student’s two-tailed *t*-test, respectively. Similarly, the proportion of participants with low HL and low eHL was analyzed using chi-squared tests. Statistical significance was determined by *p* < 0.05 except for the following categorical variables that were tested against Bonferroni adjusted alpha values: Race (0.05/3 = 0.016) and Technology Ownership (0.05/8 = 0.006) Statistical analyses were performed using STATA version 18 (StataCorp) and visualized using RStudio (Posit, PBC). Participants with missing data for survey items were included in the analytic sample.

## RESULTS

### Demographics

A total of 40 participants were enrolled (80% response rate; eFigure 1). The mean age was 35.1 ± 11.7 years. Most identified as Black (*n* = 38, 95%) and male (*n* = 23, 58%). Few participants had low HL (*n* = 17, 43%) and/or low eHL (*n* = 5, 13%). The majority (*n* = 38, 95%) of participants owned at least one technological device, with no participants having owned VR devices, and a quarter (*n* = 10, 25%) being unfamiliar with VR (Table 1).

**Table 1:** RESPITE Study Demographics

Characteristic	Interest in VR			
	**No**	**Yes**	**Total**	***p*-value**
	**(*N* = 6)**	**(*N* = 34)**	**(*N* = 40)**	
**Age (years), mean [SD]**	37.2 [11.4]	34.7 [11.8]	35.1 [11.7]	0.6^**f**^
**Gender, *n* (%)**				
Male	3 (50.0)	20 (58.8)	23 (57.5)	1.0^ g^
**Race**^**a**^**, *n* (%)**				0.8^**g**^
Black	6 (100)	32 (94.1)	38 (95.0)	
Native American	0 (0)	1 (2.9)	1 (2.5)	
Don’t know	0 (0)	1 (2.9)	1 (2.5)	
**Highest attained education, *n* (%)**				0.1^**g**^
Greater than high school	4 (66.7)	32 (94.1)	36 (90.0)	
Less than high school	2 (33.3)	2 (5.9)	4 (10.0)	
**Low health literacy (HL)**^**b**^**, *n* (%)**				
Yes	6 (100)	11 (32.4)	17 (42.5)	**0.002**^**h**^
**Low electronic health literacy (eHL)**^**c**^**, *n* (%)**				
Yes	4 (66.7)	1 (2.9)	5 (12.5)	** < 0.001**^**g**^
**Ownership of any technology device**^**d**^**, *n* (%)**				
Any^*e*^	6 (100)	32 (94.1)	38 (95.0)	0.5^**h**^
Desktop computer	0 (0)	6 (17.7)	6 (15.0)	0.3^**g**^
Laptop computer	2 (33.3)	19 (55.9)	21 (52.5)	0.4^**g**^
Tablet computer	2 (33.3)	17 (50.0)	19 (47.5)	0.7^**g**^
Cell phone (smartphone)	6 (100)	32 (94.1)	38 (95.0)	0.5^**h**^
Cell phone (not a smartphone)	0 (0)	0 (0)	0 (0)	N/A^**g**^
Video game systems	1 (16.7)	15 (44.1)	16 (40.0)	0.4^**g**^
Virtual reality tech	0 (0)	0 (0)	0 (0)	N/A^**g**^
**Familiarity with virtual reality, *n* (%)**				
Unfamiliar	3 (50.0)	7 (20.6)	10 (25.0)	0.2^ g^

^a^Bonferroni correction (0.05/3 = 0.016). ^b^Health literacy (HL) was measured through the 3-item brief health literacy screen (BHLS). The BHLS centers around filling out medical forms and independently understanding and reading materials related to health and healthcare. Low HL is defined by scoring 2 or less on any item in the BHLS. ^c^Electronic health literacy was measured through eHEALS, an 8-item survey with a composite score of 40. eHEALS assesses participants’ ability and confidence to use, value, and evaluate health resources they find using the internet. Low eHL is defined by a score of < 26, and adequate eHL is defined by a score of ≥ 26. ^d^Bonferroni correction (0.05/8 = 0.006). ^e^Ownership of any technology devices represents those participants who owned any of the following items: desktop computer, laptop computer, tablet computer, cell phone (smartphone), cell phone (not a smartphone), video game systems, or virtual reality tech. ^f^Analyzed via Student’s two-tailed *t*-test. ^g^Analyzed via Fisher’s exact test. ^h^Analyzed via chi-squared test

*N/A*, not applicable; *SD*, standard deviation

### Interests and Barriers to Using VR

Greater than three-fourths of participants indicated an interest in using VR for pain management (*n* = 34, 85%; Table 1). Participants interested in VR mostly preferred skill-development experiences, such as learning how to rock-climb and ski (*n* = 28, 85%) and adventure (*n* = 28, 85%) experiences (eFigure 2). Among participants interested in using VR, the most cited barriers to use were cost (*n* = 22, 65%) and access to VR devices (*n* = 12, 35%; eFigure 3).

### Relationship of HL and eHL with VR Interest

**Figure 1:**
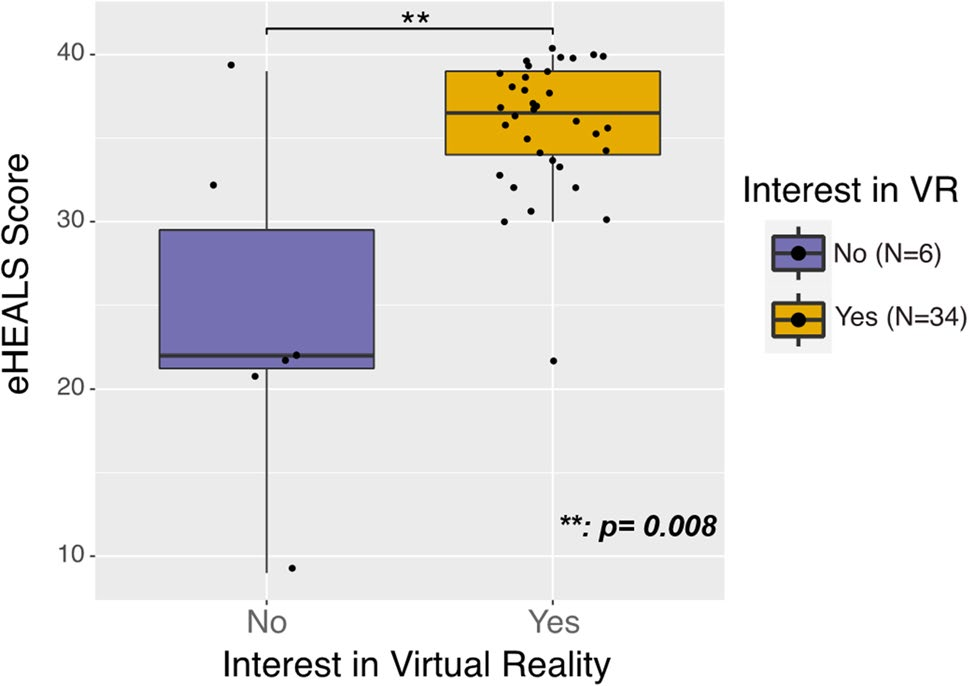
**Electronic health literacy scores by participant interest in virtual reality**

**Figure 2:**
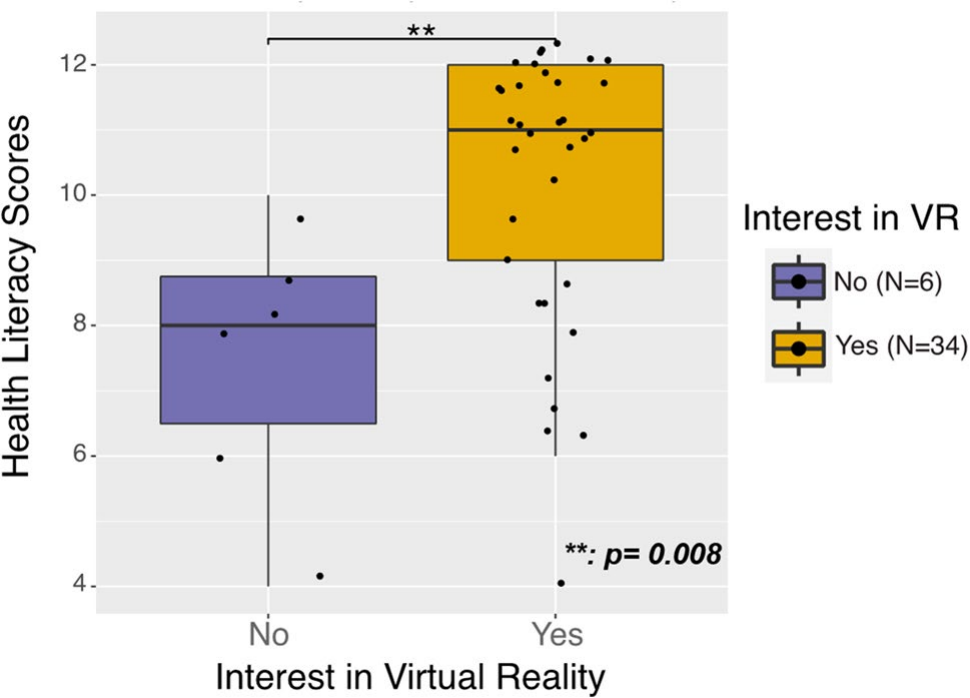
**Health literacy scores by participant interest in virtual reality**

A greater proportion of participants uninterested in using VR (versus interested) had low HL (*n* = 6, 100% vs. 11, 32%; *p* = 0.003) and low eHL (*n* = 4, 67% vs. 1, 3%; *p* < 0.001) (Table 1). Participants uninterested in using VR had lower HL (mean [95% confidence interval]: 7.5 [5.2, 9.8] vs. 10.2 [9.4–11.0];* p* = 0.008) and eHEALS (median [interquartile range]: 22, [21, 32] vs. 36.5, [34, 39];* p* = 0.008) scores than those interested (Figs. 1 and 2).

## DISCUSSION

Greater than three-fourths of adult participants with SCD were interested in using VR for pain relief, with interested participants having higher HL and eHL. Among interested participants, the majority preferred skill-development and adventure experiences and cited cost and access to VR as significant barriers to use. Our study adds to broader literature and aids in the development of VR-based interventions, with many options for genres given the wide range of interests for adults with SCD.

The high interest in VR may be explained by its novelty and experience with other distraction-based, non-pharmacological interventions to reduce pain.^19,20^ This finding is consistent with reports showing that three-quarters of patients with SCD preferred distraction-based therapies and expressed reservations over pharmacological interventions as their mainstay treatment.^7^ Yet, patients using VR for pain relief report that they would not like VR to be their sole pain management strategy.^21^ Thus, VR interventions for patients with SCD may supplement their existing pain management strategies.

Some studies report that patients value having choice over the types of VR intervention they receive for pain relief.^22^ Participants interested in VR preferred skill-development and adventure experiences, suggesting potential for tailored VR experiences. Participants cited that cost and not having access to VR were barriers for use in their home. However, these barriers may be circumvented in healthcare settings, such as use of VR in outpatient or inpatient settings if clinics or hospitals provide these devices. For instance, for pediatric patients, VR interventions have been employed in the inpatient setting.^10^ To justify the cost of VR, future studies could evaluate whether outpatient VR reduces need for hospitalization and/or whether inpatient VR interventions reduced patients’ length of stay and/or readmissions.^23^ While our population represented hospitalized patients, we assessed beyond the inpatient settings to obtain preliminary data on use of VR in outpatient settings or at home VR use for pain control.

Participants with SCD interested in VR tended to have higher HL and eHL. This is consistent with other health technology-based interventions. For instance, people with low HL are less likely to engage with health technology, including patient portals, telehealth, fitness apps, and nutrition apps.^13,24^ Similarly, we have previously found that low eHL is associated with less use of health technology, such as patient portals and telehealth.^13,15^ Our study identifies that no participants owned VR devices, and a quarter were unfamiliar with VR, which may also impact interest in eHL and HL. Perhaps the challenges of using technology experienced by people with low eHL inform attitudes toward VR. However, low HL and eHL should not exclude people from using VR; instead, they present an opportunity to design VR-based interventions that increase equitable access through education and technology support, such as educational guides, workshops, and demonstration of VR devices using electronic and general health literacy-oriented design principles to empower patients to make an informed decision in using VR for pain relief.

Study limitations include that this is a single-site study with a patient population that is predominantly urban, Black, and of lower socioeconomic status and that eligible patients were English speaking, thus limiting generalizability. Further, some of our survey instrument items may lack precision in wording (e.g., related to costs) and our survey data are self-reported and subject to participants’ recall and reporting bias. Lastly, our current study’s focus on patient preferences and interest does not capture if the intervention will reduce pain in adults with SCD. However, our study explores preferences and barriers to using VR, even hypothetically, among an understudied population, providing important insights on the patient perspective of VR for pain control.

## CONCLUSION

Over three-fourths of participants expressed interest in using VR, with HL and eHL being higher among interested participants. Skill development and adventure genres are most preferred, informing the implementation of VR-based interventions for inpatient adults with SCD. Future research should examine the feasibility, efficacy, and cost-effectiveness of VR-based interventions for reducing SCD-related pain, as well as hospital and patient perspectives on the feasibility of VR interventions using diverse options.

## Supplementary Information

Below is the link to the electronic supplementary material.

Supplementary file1 (DOCX 22 KB)

Supplementary file2 (DOCX 250 KB)

## Data Availability

The datasets generated and/or analyzed during the current study are not publicly available due to protected health information but may be made available from the corresponding author on reasonable request and with appropriate institutional approvals.
