# Supplementary material for: Virtual Reality as RESPITE: Relief Exploration for Sickle Pain Through Interventions Using Technology Engagement: A Hospital-Based Observational Study
Source: J Gen Intern Med. 2025 Aug 15;40(15):3750–4. doi: 10.1007/s11606-025-09812-z (PMC12612338; doi:10.1007/s11606-025-09812-z)
Supplement: Supplementary file 1 — Supplementary file1 (DOCX 22 KB) [file 11606_2025_9812_MOESM1_ESM.docx]

**Virtual Reality as RESPITE: Relief Exploration for Sickle Pain through Interventions using Technology Engagement: A Hospital-based Observational Study**

**Appendix: Survey Questions on Virtual Reality**

**RA Read out loud: Virtual reality –VR for short– is a simulated 3D environment that allows users to explore and interact with a realistic virtual environment. Virtual reality relies on using a headset that generates an environment that is interactive with realistic sights and sounds. With that in mind, we will be asking a few questions to explore previous experiences, if any, and preferences with VR.**

1. How familiar are you with Virtual Reality (VR)
   1. Very unfamiliar
   2. Somewhat unfamiliar
   3. Neither Familiar or Unfamiliar
   4. Somewhat familiar
   5. Very Familiar
   6. Don’t Know
   7. Refused
2. Would you be interested in using virtual reality as a way to control your pain?".
3. Yes
4. No
5. Don’t know
6. Refused
7. Virtual Reality can allow users to play games or meditatively explore worlds/surroundings. With this in mind, what sorts of Virtual Reality experiences would you be interested in? (SELECT ALL THAT APPLY)
   1. Puzzle Solving Games
   2. Adventure Games
   3. Meditation Based
   4. Skill Development (like learning how to ski, to rock climb etc)
   5. Other
   6. Don’t Know
   7. Refused
   8. Not applicable
8. What would prevent you from using VR? (SELECT ALL THAT APPLY)
   1. Cost
   2. Access at home
   3. Poor internet connection
   4. Lack of knowledge of how to use devices
   5. Lack of social support
   6. Other
   7. Don’t Know
   8. Refused
   9. Not applicable
9. What would encourage you to use VR to help manage your pain? (SELECT ALL THAT APPLY)
10. Ease of Access
11. Consistent Appointments to use devices
12. Having people who can help with technology issues
13. Presence of patient care team while using devices
14. Using VR in a multi-player setting
15. Using VR with people who experience chronic pain like my own
16. Fast Internet Connection
17. Other
18. Don’t Know
19. Refused
20. Not applicable
