# Supplementary material for: Virtual Reality as RESPITE: Relief Exploration for Sickle Pain Through Interventions Using Technology Engagement: A Hospital-Based Observational Study
Source: J Gen Intern Med. 2025 Aug 15;40(15):3750–4. doi: 10.1007/s11606-025-09812-z (PMC12612338; doi:10.1007/s11606-025-09812-z)
Supplement: Supplementary file 2 — Supplementary file2 (DOCX 250 KB) [file 11606_2025_9812_MOESM2_ESM.docx]

**Supplementary Information**

## Supplemental Figure 1: Study enrollment flow diagram from February 2023 to April 2024

## Supplemental Figure 2: Preferred Virtual Reality Experiences Among Participants Interested in Virtual Reality. Bar charts depicting preferred virtual reality experiences among participants interested in using virtual reality (N=34). Maroon color indicates experience with highest preference among participants.

## Supplemental Figure 3: Barriers to Using Virtual Reality Among Participants Interested in Virtual Reality. Bar charts depicting barriers to using virtual reality among participants interested in using virtual reality (N=34). Maroon color indicates experience with highest number of participants citing this barrier. Technology issues represents lack of knowledge to use such devices. “Other” category also includes “Don’t Know” (n=2) and “Not Applicable” (n=7).

## Supplemental Figure 1

## Supplemental Figure 2

## Supplemental Figure 3
